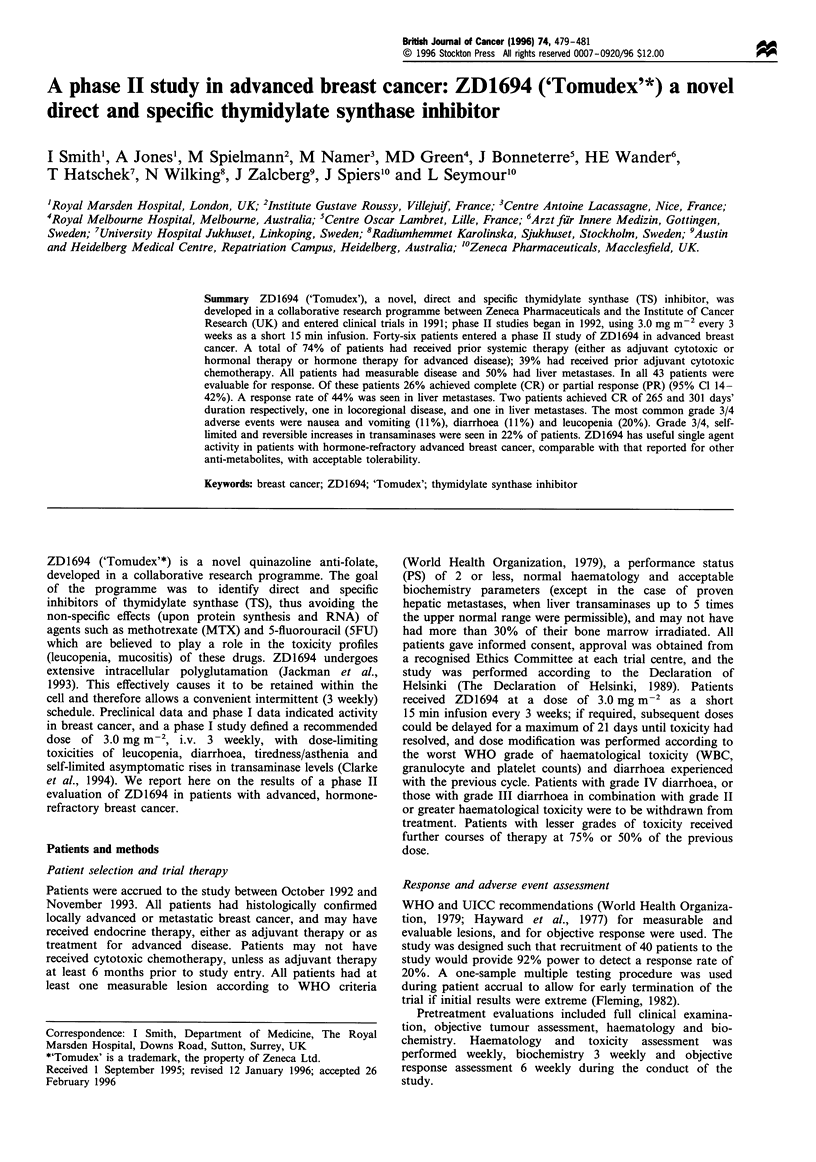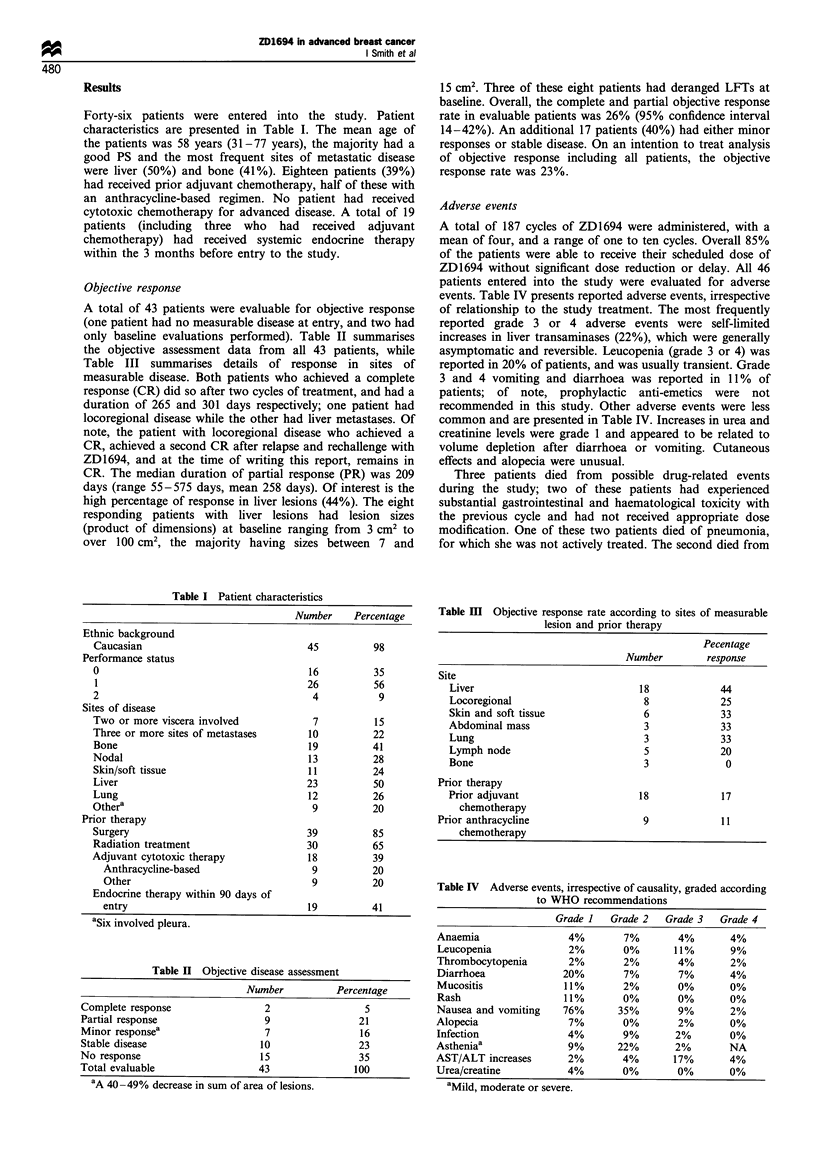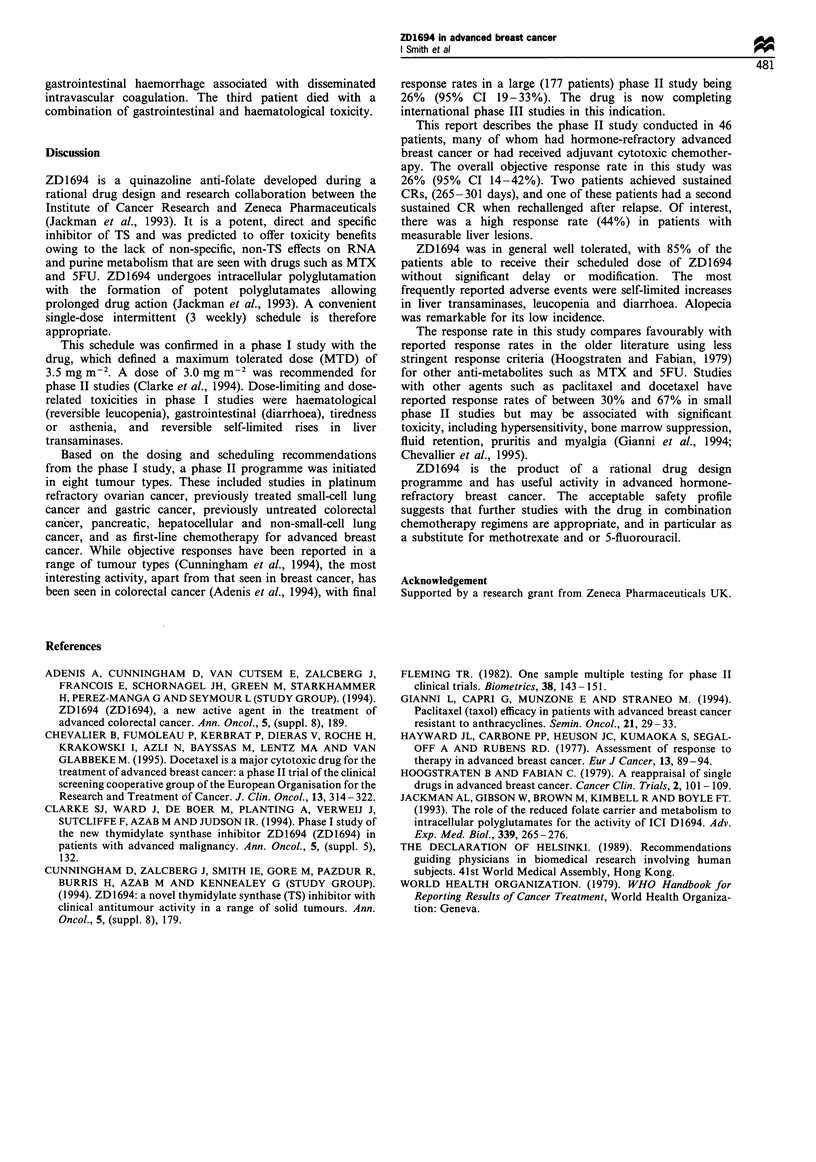# A phase II study in advanced breast cancer: ZD1694 ('Tomudex') a novel direct and specific thymidylate synthase inhibitor.

**DOI:** 10.1038/bjc.1996.386

**Published:** 1996-08

**Authors:** I. Smith, A. Jones, M. Spielmann, M. Namer, M. D. Green, J. Bonneterre, H. E. Wander, T. Hatschek, N. Wilking, J. Zalcberg, J. Spiers, L. Seymour

**Affiliations:** Royal Marsden Hospital, London, UK.

## Abstract

ZD1694 ('Tomudex'), a novel, direct and specific thymidylate synthase (TS) inhibitor, was developed in a collaborative research programme between Zeneca Pharmaceuticals and the Institute of Cancer Research (UK) and entered clinical trials in 1991; phase II studies began in 1992, using 3.0 mg m-2 every 3 weeks as a short 15 min infusion. Forty-six patients entered a phase II study of ZD1694 in advanced breast cancer. A total of 74% of patients had received prior systemic therapy (either as adjuvant cytotoxic or hormonal therapy or hormone therapy for advanced disease); 39% had received prior adjuvant cytotoxic chemotherapy. All patients had measurable disease and 50% had liver metastases. In all 43 patients were evaluable for response. Of these patients 26% achieved complete (CR) or partial response (PR) (95% Cl 14-42%). A response rate of 44% was seen in liver metastases. Two patients achieved CR of 265 and 301 days' duration respectively, one in locoregional disease, and one in liver metastases. The most common grade 3/4 adverse events were nausea and vomiting (11%), diarrhoea (11%) and leucopenia (20%). Grade 3/4, self-limited and reversible increases in transaminases were seen in 22% of patients. ZD1694 has useful single agent activity in patients with hormone-refractory advanced breast cancer, comparable with that reported for other anti-metabolites, with acceptable tolerability.


British Journal of Cancer (1996) 74, 479-481
© 1996 Stockton Press All rights reserved 0007-0920/96 $12.00

### A phase II study in advanced breast cancer: ZD1694 ('Tomudex'*) a novel direct and specific thymidylate synthase inhibitor

I Smith[1], A Jones[1], M Spielmann[2], M Namer[3], MD Green[4], J Bonneterre[5], HE Wander[6], T Hatschek[7], N Wilking[8], J Zalcberg[9], J Spiers[10] and L Seymour[10]

*[1]Royal Marsden Hospital, London, UK; [2]Institute Gustave Roussy, Villejuif, France; [3]Centre Antoine Lacassagne, Nice, France; [4]Royal Melbourne Hospital, Melbourne, Australia; [5]Centre Oscar Lambret, Lille, France; [6]Arzt für Innere Medizin, Gottingen, Sweden; [7]University Hospital Jukhuset, Linkoping, Sweden; [8]Radiumhemmet Karolinska, Sjukhuset, Stockholm, Sweden; [9]Austin and Heidelberg Medical Centre, Repatriation Campus, Heidelberg, Australia; [10]Zeneca Pharmaceuticals, Macclesfield, UK.*

**Summary** ZD1694 ('Tomudex'), a novel, direct and specific thymidylate synthase (TS) inhibitor, was developed in a collaborative research programme between Zeneca Pharmaceuticals and the Institute of Cancer Research (UK) and entered clinical trials in 1991; phase II studies began in 1992, using 3.0 mg m$^{-2}$ every 3 weeks as a short 15 min infusion. Forty-six patients entered a phase II study of ZD1694 in advanced breast cancer. A total of 74% of patients had received prior systemic therapy (either as adjuvant cytotoxic or hormonal therapy or hormone therapy for advanced disease); 39% had received prior adjuvant cytotoxic chemotherapy. All patients had measurable disease and 50% had liver metastases. In all 43 patients were evaluable for response. Of these patients 26% achieved complete (CR) or partial response (PR) (95% Cl 14–42%). A response rate of 44% was seen in liver metastases. Two patients achieved CR of 265 and 301 days' duration respectively, one in locoregional disease, and one in liver metastases. The most common grade 3/4 adverse events were nausea and vomiting (11%), diarrhoea (11%) and leucopenia (20%). Grade 3/4, self-limited and reversible increases in transaminases were seen in 22% of patients. ZD1694 has useful single agent activity in patients with hormone-refractory advanced breast cancer, comparable with that reported for other anti-metabolites, with acceptable tolerability.

**Keywords:** breast cancer; ZD1694; 'Tomudex'; thymidylate synthase inhibitor

ZD1694 ('Tomudex'*) is a novel quinazoline anti-folate, developed in a collaborative research programme. The goal of the programme was to identify direct and specific inhibitors of thymidylate synthase (TS), thus avoiding the non-specific effects (upon protein synthesis and RNA) of agents such as methotrexate (MTX) and 5-fluorouracil (5FU) which are believed to play a role in the toxicity profiles (leucopenia, mucositis) of these drugs. ZD1694 undergoes extensive intracellular polyglutamation (Jackman *et al.*, 1993). This effectively causes it to be retained within the cell and therefore allows a convenient intermittent (3 weekly) schedule. Preclinical data and phase I data indicated activity in breast cancer, and a phase I study defined a recommended dose of 3.0 mg m$^{-2}$, i.v. 3 weekly, with dose-limiting toxicities of leucopenia, diarrhoea, tiredness/asthenia and self-limited asymptomatic rises in transaminase levels (Clarke *et al.*, 1994). We report here on the results of a phase II evaluation of ZD1694 in patients with advanced, hormone-refractory breast cancer.

### Patients and methods

### *Patient selection and trial therapy*

Patients were accrued to the study between October 1992 and November 1993. All patients had histologically confirmed locally advanced or metastatic breast cancer, and may have received endocrine therapy, either as adjuvant therapy or as treatment for advanced disease. Patients may not have received cytotoxic chemotherapy, unless as adjuvant therapy at least 6 months prior to study entry. All patients had at least one measurable lesion according to WHO criteria (World Health Organization, 1979), a performance status (PS) of 2 or less, normal haematology and acceptable biochemistry parameters (except in the case of proven hepatic metastases, when liver transaminases up to 5 times the upper normal range were permissible), and may not have had more than 30% of their bone marrow irradiated. All patients gave informed consent, approval was obtained from a recognised Ethics Committee at each trial centre, and the study was performed according to the Declaration of Helsinki (The Declaration of Helsinki, 1989). Patients received ZD1694 at a dose of 3.0 mg m$^{-2}$ as a short 15 min infusion every 3 weeks; if required, subsequent doses could be delayed for a maximum of 21 days until toxicity had resolved, and dose modification was performed according to the worst WHO grade of haematological toxicity (WBC, granulocyte and platelet counts) and diarrhoea experienced with the previous cycle. Patients with grade IV diarrhoea, or those with grade III diarrhoea in combination with grade II or greater haematological toxicity were to be withdrawn from treatment. Patients with lesser grades of toxicity received further courses of therapy at 75% or 50% of the previous dose.

### *Response and adverse event assessment*

WHO and UICC recommendations (World Health Organization, 1979; Hayward *et al.*, 1977) for measurable and evaluable lesions, and for objective response were used. The study was designed such that recruitment of 40 patients to the study would provide 92% power to detect a response rate of 20%. A one-sample multiple testing procedure was used during patient accrual to allow for early termination of the trial if initial results were extreme (Fleming, 1982).

Pretreatment evaluations included full clinical examination, objective tumour assessment, haematology and biochemistry. Haematology and toxicity assessment was performed weekly, biochemistry 3 weekly and objective response assessment 6 weekly during the conduct of the study.

Correspondence: I Smith, Department of Medicine, The Royal Marsden Hospital, Downs Road, Sutton, Surrey, UK
*'Tomudex' is a trademark, the property of Zeneca Ltd.
Received 1 September 1995; revised 12 January 1996; accepted 26 February 1996

### Results

Forty-six patients were entered into the study. Patient characteristics are presented in Table I. The mean age of the patients was 58 years (31–77 years), the majority had a good PS and the most frequent sites of metastatic disease were liver (50%) and bone (41%). Eighteen patients (39%) had received prior adjuvant chemotherapy, half of these with an anthracycline-based regimen. No patient had received cytotoxic chemotherapy for advanced disease. A total of 19 patients (including three who had received adjuvant chemotherapy) had received systemic endocrine therapy within the 3 months before entry to the study.

### *Objective response*

A total of 43 patients were evaluable for objective response (one patient had no measurable disease at entry, and two had only baseline evaluations performed). Table II summarises the objective assessment data from all 43 patients, while Table III summarises details of response in sites of measurable disease. Both patients who achieved a complete response (CR) did so after two cycles of treatment, and had a duration of 265 and 301 days respectively; one patient had locoregional disease while the other had liver metastases. Of note, the patient with locoregional disease who achieved a CR, achieved a second CR after relapse and rechallenge with ZD1694, and at the time of writing this report, remains in CR. The median duration of partial response (PR) was 209 days (range 55–575 days, mean 258 days). Of interest is the high percentage of response in liver lesions (44%). The eight responding patients with liver lesions had lesion sizes (product of dimensions) at baseline ranging from 3 $cm^2$ to over 100 $cm^2$, the majority having sizes between 7 and 15 $cm^2$. Three of these eight patients had deranged LFTs at baseline. Overall, the complete and partial objective response rate in evaluable patients was 26% (95% confidence interval 14–42%). An additional 17 patients (40%) had either minor responses or stable disease. On an intention to treat analysis of objective response including all patients, the objective response rate was 23%.

### *Adverse events*

A total of 187 cycles of ZD1694 were administered, with a mean of four, and a range of one to ten cycles. Overall 85% of the patients were able to receive their scheduled dose of ZD1694 without significant dose reduction or delay. All 46 patients entered into the study were evaluated for adverse events. Table IV presents reported adverse events, irrespective of relationship to the study treatment. The most frequently reported grade 3 or 4 adverse events were self-limited increases in liver transaminases (22%), which were generally asymptomatic and reversible. Leucopenia (grade 3 or 4) was reported in 20% of patients, and was usually transient. Grade 3 and 4 vomiting and diarrhoea was reported in 11% of patients; of note, prophylactic anti-emetics were not recommended in this study. Other adverse events were less common and are presented in Table IV. Increases in urea and creatinine levels were grade 1 and appeared to be related to volume depletion after diarrhoea or vomiting. Cutaneous effects and alopecia were unusual.

Three patients died from possible drug-related events during the study; two of these patients had experienced substantial gastrointestinal and haematological toxicity with the previous cycle and had not received appropriate dose modification. One of these two patients died of pneumonia, for which she was not actively treated. The second died from

**Table I** Patient characteristics

| | *Number* | *Percentage* |
|---|---|---|
| Ethnic background | | |
| Caucasian | 45 | 98 |
| Performance status | | |
| 0 | 16 | 35 |
| 1 | 26 | 56 |
| 2 | 4 | 9 |
| Sites of disease | | |
| Two or more viscera involved | 7 | 15 |
| Three or more sites of metastases | 10 | 22 |
| Bone | 19 | 41 |
| Nodal | 13 | 28 |
| Skin/soft tissue | 11 | 24 |
| Liver | 23 | 50 |
| Lung | 12 | 26 |
| Other[a] | 9 | 20 |
| Prior therapy | | |
| Surgery | 39 | 85 |
| Radiation treatment | 30 | 65 |
| Adjuvant cytotoxic therapy | 18 | 39 |
| Anthracycline-based | 9 | 20 |
| Other | 9 | 20 |
| Endocrine therapy within 90 days of entry | 19 | 41 |

[a]Six involved pleura.

**Table II** Objective disease assessment

| | *Number* | *Percentage* |
|---|---|---|
| Complete response | 2 | 5 |
| Partial response | 9 | 21 |
| Minor response[a] | 7 | 16 |
| Stable disease | 10 | 23 |
| No response | 15 | 35 |
| Total evaluable | 43 | 100 |

[a]A 40–49% decrease in sum of area of lesions.

**Table III** Objective response rate according to sites of measurable lesion and prior therapy

| | *Number* | *Pecentage response* |
|---|---|---|
| Site | | |
| Liver | 18 | 44 |
| Locoregional | 8 | 25 |
| Skin and soft tissue | 6 | 33 |
| Abdominal mass | 3 | 33 |
| Lung | 3 | 33 |
| Lymph node | 5 | 20 |
| Bone | 3 | 0 |
| Prior therapy | | |
| Prior adjuvant chemotherapy | 18 | 17 |
| Prior anthracycline chemotherapy | 9 | 11 |

**Table IV** Adverse events, irrespective of causality, graded according to WHO recommendations

| | *Grade 1* | *Grade 2* | *Grade 3* | *Grade 4* |
|---|---|---|---|---|
| Anaemia | 4% | 7% | 4% | 4% |
| Leucopenia | 2% | 0% | 11% | 9% |
| Thrombocytopenia | 2% | 2% | 4% | 2% |
| Diarrhoea | 20% | 7% | 7% | 4% |
| Mucositis | 11% | 2% | 0% | 0% |
| Rash | 11% | 0% | 0% | 0% |
| Nausea and vomiting | 76% | 35% | 9% | 2% |
| Alopecia | 7% | 0% | 2% | 0% |
| Infection | 4% | 9% | 2% | 0% |
| Asthenia[a] | 9% | 22% | 2% | NA |
| AST/ALT increases | 2% | 4% | 17% | 4% |
| Urea/creatine | 4% | 0% | 0% | 0% |

[a]Mild, moderate or severe.

gastrointestinal haemorrhage associated with disseminated intravascular coagulation. The third patient died with a combination of gastrointestinal and haematological toxicity.

### Discussion

ZD1694 is a quinazoline anti-folate developed during a rational drug design and research collaboration between the Institute of Cancer Research and Zeneca Pharmaceuticals (Jackman *et al.*, 1993). It is a potent, direct and specific inhibitor of TS and was predicted to offer toxicity benefits owing to the lack of non-specific, non-TS effects on RNA and purine metabolism that are seen with drugs such as MTX and 5FU. ZD1694 undergoes intracellular polyglutamation with the formation of potent polyglutamates allowing prolonged drug action (Jackman *et al.*, 1993). A convenient single-dose intermittent (3 weekly) schedule is therefore appropriate.

This schedule was confirmed in a phase I study with the drug, which defined a maximum tolerated dose (MTD) of 3.5 mg $m^{-2}$. A dose of 3.0 mg $m^{-2}$ was recommended for phase II studies (Clarke *et al.*, 1994). Dose-limiting and dose-related toxicities in phase I studies were haematological (reversible leucopenia), gastrointestinal (diarrhoea), tiredness or asthenia, and reversible self-limited rises in liver transaminases.

Based on the dosing and scheduling recommendations from the phase I study, a phase II programme was initiated in eight tumour types. These included studies in platinum refractory ovarian cancer, previously treated small-cell lung cancer and gastric cancer, previously untreated colorectal cancer, pancreatic, hepatocellular and non-small-cell lung cancer, and as first-line chemotherapy for advanced breast cancer. While objective responses have been reported in a range of tumour types (Cunningham *et al.*, 1994), the most interesting activity, apart from that seen in breast cancer, has been seen in colorectal cancer (Adenis *et al.*, 1994), with final response rates in a large (177 patients) phase II study being 26% (95% CI 19–33%). The drug is now completing international phase III studies in this indication.

This report describes the phase II study conducted in 46 patients, many of whom had hormone-refractory advanced breast cancer or had received adjuvant cytotoxic chemotherapy. The overall objective response rate in this study was 26% (95% CI 14–42%). Two patients achieved sustained CRs, (265–301 days), and one of these patients had a second sustained CR when rechallenged after relapse. Of interest, there was a high response rate (44%) in patients with measurable liver lesions.

ZD1694 was in general well tolerated, with 85% of the patients able to receive their scheduled dose of ZD1694 without significant delay or modification. The most frequently reported adverse events were self-limited increases in liver transaminases, leucopenia and diarrhoea. Alopecia was remarkable for its low incidence.

The response rate in this study compares favourably with reported response rates in the older literature using less stringent response criteria (Hoogstraten and Fabian, 1979) for other anti-metabolites such as MTX and 5FU. Studies with other agents such as paclitaxel and docetaxel have reported response rates of between 30% and 67% in small phase II studies but may be associated with significant toxicity, including hypersensitivity, bone marrow suppression, fluid retention, pruritis and myalgia (Gianni *et al.*, 1994; Chevallier *et al.*, 1995).

ZD1694 is the product of a rational drug design programme and has useful activity in advanced hormone-refractory breast cancer. The acceptable safety profile suggests that further studies with the drug in combination chemotherapy regimens are appropriate, and in particular as a substitute for methotrexate and or 5-fluorouracil.

**Acknowledgement**
Supported by a research grant from Zeneca Pharmaceuticals UK.

### References

ADENIS A, CUNNINGHAM D, VAN CUTSEM E, ZALCBERG J, FRANCOIS E, SCHORNAGEL JH, GREEN M, STARKHAMMER H, PEREZ-MANGA G AND SEYMOUR L (STUDY GROUP). (1994). ZD1694 (ZD1694), a new active agent in the treatment of advanced colorectal cancer. *Ann. Oncol.*, **5**, (suppl. 8), 189.

CHEVALIER B, FUMOLEAU P, KERBRAT P, DIERAS V, ROCHE H, KRAKOWSKI I, AZLI N, BAYSSAS M, LENTZ MA AND VAN GLABBEKE M. (1995). Docetaxel is a major cytotoxic drug for the treatment of advanced breast cancer: a phase II trial of the clinical screening cooperative group of the European Organisation for the Research and Treatment of Cancer. *J. Clin. Oncol.*, **13**, 314–322.

CLARKE SJ, WARD J, DE BOER M, PLANTING A, VERWEIJ J, SUTCLIFFE F, AZAB M AND JUDSON IR. (1994). Phase I study of the new thymidylate synthase inhibitor ZD1694 (ZD1694) in patients with advanced malignancy. *Ann. Oncol.*, **5**, (suppl. 5), 132.

CUNNINGHAM D, ZALCBERG J, SMITH IE, GORE M, PAZDUR R, BURRIS H, AZAB M AND KENNEALEY G (STUDY GROUP). (1994). ZD1694: a novel thymidylate synthase (TS) inhibitor with clinical antitumour activity in a range of solid tumours. *Ann. Oncol.*, **5**, (suppl. 8), 179.

FLEMING TR. (1982). One sample multiple testing for phase II clinical trials. *Biometrics*, **38**, 143–151.

GIANNI L, CAPRI G, MUNZONE E AND STRANEO M. (1994). Paclitaxel (taxol) efficacy in patients with advanced breast cancer resistant to anthracyclines. *Semin. Oncol.*, **21**, 29–33.

HAYWARD JL, CARBONE PP, HEUSON JC, KUMAOKA S, SEGALOFF A AND RUBENS RD. (1977). Assessment of response to therapy in advanced breast cancer. *Eur J Cancer*, **13**, 89–94.

HOOGSTRATEN B AND FABIAN C. (1979). A reappraisal of single drugs in advanced breast cancer. *Cancer Clin. Trials*, **2**, 101–109.

JACKMAN AL, GIBSON W, BROWN M, KIMBELL R AND BOYLE FT. (1993). The role of the reduced folate carrier and metabolism to intracellular polyglutamates for the activity of ICI D1694. *Adv. Exp. Med. Biol.*, **339**, 265–276.

THE DECLARATION OF HELSINKI. (1989). Recommendations guiding physicians in biomedical research involving human subjects. 41st World Medical Assembly, Hong Kong.

WORLD HEALTH ORGANIZATION. (1979). *WHO Handbook for Reporting Results of Cancer Treatment*, World Health Organization: Geneva.